# Identification and Regulation of Tomato Serine/Arginine-Rich Proteins Under High Temperatures

**DOI:** 10.3389/fpls.2021.645689

**Published:** 2021-03-29

**Authors:** Remus R. E. Rosenkranz, Samia Bachiri, Stavros Vraggalas, Mario Keller, Stefan Simm, Enrico Schleiff, Sotirios Fragkostefanakis

**Affiliations:** ^1^Department of Biosciences, Molecular Cell Biology of Plants, Goethe University, Frankfurt am Main, Germany; ^2^Buchmann Institute for Molecular Life Sciences, Goethe University, Frankfurt am Main, Germany; ^3^Institute of Bioinformatics, University Medicine Greifswald, Greifswald, Germany; ^4^Frankfurt Institute of Advanced Studies, Frankfurt am Main, Germany

**Keywords:** alternative splicing, pre-mRNA, heat stress, *Solanum lycopersicum*, regulation

## Abstract

Alternative splicing is an important mechanism for the regulation of gene expression in eukaryotes during development, cell differentiation or stress response. Alterations in the splicing profiles of genes under high temperatures that cause heat stress (HS) can impact the maintenance of cellular homeostasis and thermotolerance. Consequently, information on factors involved in HS-sensitive alternative splicing is required to formulate the principles of HS response. Serine/arginine-rich (SR) proteins have a central role in alternative splicing. We aimed for the identification and characterization of SR-coding genes in tomato (*Solanum lycopersicum*), a plant extensively used in HS studies. We identified 17 canonical SR and two SR-like genes. Several SR-coding genes show differential expression and altered splicing profiles in different organs as well as in response to HS. The transcriptional induction of five SR and one SR-like genes is partially dependent on the master regulator of HS response, HS transcription factor HsfA1a. *Cis*-elements in the promoters of these SR genes were predicted, which can be putatively recognized by HS-induced transcription factors. Further, transiently expressed SRs show reduced or steady-state protein levels in response to HS. Thus, the levels of SRs under HS are regulated by changes in transcription, alternative splicing and protein stability. We propose that the accumulation or reduction of SRs under HS can impact temperature-sensitive alternative splicing.

## Introduction

Alternative splicing is an important regulatory mechanism in eukaryotes, contributing to the increase of proteome diversity and regulation of protein abundance (Reddy et al., 2013; Staiger and Brown, 2013). More than 40% of intron-containing genes in *Arabidopsis thaliana* (Filichkin et al., 2010), *Zea mays* (Thatcher et al., 2014), and *Oryza sativa* (Lu et al., 2010) are subjected to alternative splicing. In plants, intron retention is the most prevalent alternative splicing event (Wang and Brendel, 2006). However, a substantial number of genes undergo other types of alternative splicing, such as exon skipping or selection of alternative donor or acceptor sites (Ner-Gaon et al., 2004; Reddy, 2007; Filichkin et al., 2010; Lu et al., 2010; Jiang et al., 2017; Keller et al., 2017). The selection of splice sites in many pre-mRNAs is conditional and dependent on cell-type, developmental stage and environmental conditions (Filichkin et al., 2015). Consequently, factors regulating alternative splicing are key players in development and stress responses (Staiger and Brown, 2013; Szakonyi and Duque, 2018).

Serine/arginine-rich proteins (SR) are core regulators of constitutive and alternative pre-mRNA splicing, but recent studies suggest additional functions in the regulation of mRNA export, stability and translation (Howard and Sanford, 2015). While the human SR family consists of 12 members (Manley and Krainer, 2010), plants encode for a higher number of SR genes with 18 members in *A. thaliana*, 25 in *Brassica rapa*, 22 in *Oryza sativa*, and 40 *Triticum aestivum* (Isshiki et al., 2006; Barta et al., 2010; Richardson et al., 2011; Chen et al., 2019). The expansion of the SR gene family in plants resulted in the emergence of SR proteins with additional domains and features (Barta et al., 2010; Califice et al., 2011).

Canonical SRs have one or two amino-terminal RNA-recognition motifs (RRM), followed by a serine-arginine dipeptide-rich carboxyl-terminal region involved in both protein-RNA and protein-protein interactions (Barta et al., 2010). Plant SRs are categorized into six subfamilies based on their modular organization and the presence of amino acid sequence motifs (Barta et al., 2010; Richardson et al., 2011). The SC subfamily contains orthologs of the mammalian SC35 protein that comprise a single RRM followed by an RS domain. SC-like (SCL) proteins form a distinct subfamily found only in plants with a characteristic charged N-terminal extension. The SR subfamily contains orthologs of the mammalian SRSF1 with a second RRM possessing a conserved SWQDLKD motif (Barta et al., 2010). In some plant SRs, this motif does not exist. These SRs have been assigned to the plant-specific RS subfamily. The RSZ subfamily contains orthologs of the mammalian SRSF7 with a single RRM as well as a zinc knuckle (ZnK) domain. SR proteins with two ZnK domains make the RS2Z subfamily (Barta et al., 2010).

SRs are regulated at multiple levels contributing to their transcript and protein control, subcellular localization and function (Cazalla et al., 2002; Ali et al., 2003; Cruz et al., 2014; Hartmann et al., 2018). The majority of SR genes themselves undergo alternative splicing (Palusa et al., 2007). Unproductive splicing results in aberrant mRNAs that get depleted by non-sense mRNA decay (NMD; Palusa and Reddy, 2010). Putative protein-coding splice variants exist as well, which exhibit a range of variations when compared to the full-length protein (Palusa et al., 2007). At-SR34a and At-RS41 isoforms are predicted to differ from the canonically spliced variant in only three and two amino acid residues, respectively. In other cases, isoforms are predicted to encode for putative proteins with truncated or entirely missing domains (Palusa et al., 2007). Interestingly, for some SRs, alternative splicing occurs in an autoregulatory manner as shown for At-SCL33 and At-SR30 (Thomas et al., 2012; Hartmann et al., 2018).

Tomato is an economically important crop and model plant for the analysis of fleshy fruit development and abiotic stress responses, including heat stress (HS; Mishra et al., 2002; Frank et al., 2009; Giorno et al., 2010; Tomato Genome Consortium, 2012; Fragkostefanakis et al., 2016; Müller et al., 2016; Keller et al., 2017). At the transcriptional level, HS response is mainly controlled by a network of HS transcription factors (Hsf; Scharf et al., 2012). In tomato, HsfA1a is the master regulator of HS response and thermotolerance (Mishra et al., 2002). It induces the expression of other Hsfs such as HsfA2, and thereby allows the formation of hetero-oligomeric complexes with strong transactivation activity that leads to the further upregulation of HS genes (Scharf et al., 1998). HsfA2, the HSF important for HS acclimation, is, among others, also affected by alternative splicing in both tomato and *A. thaliana* (Keller et al., 2017; Hu et al., 2020b). Therefore, it is evident that alternative splicing contributes to HS response regulation and thermotolerance. Consequently, the identification of factors involved in HS-related alternative splicing events is required to complement the model on regulatory mechanisms under elevated temperatures.

Here, as a first step toward understanding the regulation of alternative splicing in tomato we aimed to identify and characterize the tomato SR protein family. Following the identification of tomato SR-coding genes, we examined their transcript abundance and splicing profile under control conditions in different tissues, as well as in heat stressed leaves, as differential expression can be indicative of preferential activity. We show that high temperature impacts SR coding genes and proteins at multiple levels in a distinct manner, even among members of the same subfamily, pointing to distinct roles of individual members in pre-mRNA processing under high temperatures.

## Materials and Methods

### Plant Material and Stress Treatments

HS treatments were performed in excised leaves from 6-week old plants of *Solanum lycopersicum* (cv. Moneymaker) wild-type. Leaves were placed in parafilm-sealed Petri dishes in water baths at the appropriate temperature. In addition, we used the previously characterized transgenic A1CS plants showing HsfA1a co-suppression (CS) due to the presence of a tandem inverted repeat of HsfA1a expression cassette, leading to the RNAi-mediated posttranscriptional gene silencing (Mishra et al., 2002). For expression analysis in different tomato tissues, we used the roots, the two upper internodes of the stem and 3 young leaves from 6 week old wild-type plants grown in ½ strength Murashige and Skoog (MS) (Murashige and Skoog, 1962) medium in 1 liter pots under a 16 h/25°C day and 8 h/22°C night cycle. In addition, pooled flower buds having anthers of at least 6 mm length till the stage of anthesis (FL), immature green (IG) fruits having 50% the size of mature fruits, as well as fruits at mature green (MG), breaker (BR), turning (TU) and red ripe (RR) stages from 2 to 3 month old wild-type plants were used. Samples included tissues from different plants grown in a greenhouse in pots with soil under a 16 h/25°C day and 8 h/22°C night cycle.

### RNA Extraction and Transcript Profiling

Total RNA was extracted from young tomato leaves or tomato mesophyll protoplasts using the E.Z.N.A. Plant RNA kit (Omega Bio-Tek) following the guidelines of the manufacturer and subsequently treated with DNaseI. One microgram of DNA-free RNA was used for cDNA synthesis using the reverse transcriptase RevertAid (ThermoFisher). Splicing profile of each SR was done via PCR using oligonucleotide primers flanking the alternatively spliced sites of each gene within the open reading frame (ORF). Reactions were run for 30 cycles for all SRs, with the exception of Sl-RS30 and Sl-SR33 which were run for 32 cycles and Sl-SCL29 and Sl-SR35 for 28 cycles. Reactions were analyzed by agarose gel electrophoresis after ethidium bromide staining and imaging on a UV transilluminator using a CCD camera. Signal intensities were quantified using ImageJ 1.53 (Schindelin et al., 2012). Splicing efficiency was calculated based on the fraction of protein-coding transcripts as percentage of the total signal derived from all splice variants. Two biological replicates were used for analysis of splicing profile in different tissues and three independent replicates for analysis of splicing under HS. –RT reactions confirmed the absence of genomic DNA in the samples.

Quantitative real-time PCR (qRT-PCR) was performed on a StepOnePlus cycler (ThermoFischer) to investigate relative transcript levels of different genes in cDNA samples. A SYBR mix PowerUp (ThermoFisher) was used for PCR, with 0.3 μM of each oligonucleotide. Thermal cycling conditions were 50°C for 2 min, followed by 95°C/3 min, and then 40 cycles of 95°C/15 s, 60°C/30 s, 72°C/30 s. Gene oligonucleotides (Supplementary Table 1) were designed using PRIMER3 (https://bioinfo.ut.ee/primer3-0.4.0/). EF1α (Solyc06g005060) and CAC (Solyc08g006960) served as an internal control for developmental expression and EF1α for HS. Fold changes were calculated based on the 2^−ΔΔCt^ method (Livak and Schmittgen, 2001). Three biological replicates were used for the analysis.

### Transient Expression of SR Proteins in Mesophyll Protoplasts

*S. lycopersicum* cv. Moneymaker mesophyll protoplasts were isolated and transformed by polyethylene glycol (PEG)-mediated transformation (Mishra et al., 2002). In short, 50,000 protoplasts were transfected with plasmid DNA (10 μg total) carrying the desired expression cassette. pRT-Neo plasmid carrying a neomycin phosphotransferase gene was used as mock control and to adjust total DNA amounts. pRT plasmids carrying the HsfA1a and HsfA2 expression cassettes have been previously described (Scharf et al., 1998). For the expression of HA-tagged SR proteins, the CDS of the respective full-length protein-coding transcript (splice variant 1) was amplified using oligonucleotides described in Supplementary Table 1 and subsequently cloned into a pRT plasmid using cDNA from leaves. The expression vectors used are derivatives of pRT101 (Töpfer et al., 1988). In all cases, the CDS of the respective gene is under the control of the CaMV 35S promoter. Restriction sites used for cloning are described in Supplementary Table 1 as well. For expression, protoplasts were incubated for 4–5 h at 25°C and then exposed to the indicated temperatures in water baths for 1 h. Three biological replicates were used for the analysis.

### Immunodetection Analysis

Protein extracts (15–20 μg) were separated on 10% SDS-polyacrylamide gels. For immunoblot analysis, proteins were transferred to a nitrocellulose membrane (PROTRAN® Nitrocellulose Transfer Membrane, Whatman) and signals were obtained via chemiluminescence detection following the manufacturer's protocol (PerkinElmer). Anti-HA antibodies were used for detection of HA-tagged SR proteins as described (Mishra et al., 2002). As internal control we detected the endogenous thermostable heat shock cognate 70 kDa protein (Hsc70) by an anti-Hsc70 antibody (Mishra et al., 2002). Signal intensities were quantified using ImageJ 1.53 (Schindelin et al., 2012) and normalized to Hsc70. Three biological replicates were used for the analysis.

### Identification of Putative Tomato SR Proteins and Motif Analysis

For the identification of SR orthologous groups in *S. lycopersicum*, the SR proteins inventory from *Arabidopsis thaliana, Oryza sativa, Sorghum bicolor* and *Zea mays* SRs as baits (Chen, 2006; Richardson et al., 2011) was used. An ortholog search was performed based on OrthoMCL (Chen, 2006) and InParanoid (Östlund et al., 2009). Furthermore, a reciprocal best-BLAST hit search was used to add a more lose ortholog search to prevent the missing of false negatives. Additional SR proteins were identified by whole genome search on protein models (ITAG 2.5) based on the guidelines for plant SRs (Barta et al., 2010). Amino acid sequence motifs were identified via MEME tool (Bailey et al., 2015), (*E*-value < 0.1). The phylogenetic tree of Arabidopsis and tomato SR proteins was constructed using Phylogeny.fr software and visualized using iTOL.

### Data Collection and Alignment of RNA-Seq Datasets

RNA-seq datasets from *S. lycopersicum cv*. Moneymaker were downloaded from the European Nucleotide Archive (ENA, www.ebi.ac.uk/ena) for fruit (ERR426391 and ERR426393) and root (ERR1533156, ERR1533157, and ERR1533158) as well as from the Gene Expression Omnibus (GEO, www.ncbi.nlm.nih.gov/geo/) for pollen (GSM2131175 and GSM2131176). Reads from biological replicates were merged and aligned to the *S. lycopersicum* reference genome (ITAG2.4, cv. Heinz) available at the Sol Genomics Network (SGN, www.solgenomics.net) using HISAT2 (version 2.0.4, Pertea et al., 2016) in either single-end (fruit) or paired-end mode (root and pollen) with default parameters.

### Reconstruction and Quantification of SR Transcripts

HISAT2 alignments of merged RNA-seq runs from pollen, fruit and root were independently used for the reconstruction of putative transcript isoforms of SR proteins. The reconstruction was based on reads aligning to known (ITAG2.4) and novel manually revised SR protein loci. A revision of the ITAG2.4 annotations was conducted by elongation based on reads that aligned beyond gene boundaries, revealing not annotated exons outside the current annotation. The reconstruction of SR protein isoforms was performed by: (i) Spliced reads were used for the determination and quantification of splice junctions. These reads comprise parts of two or more exonic regions. By this, the position of splice junctions can be inferred. For complexity reduction, initially the splice junctions with an abundance of <2.5% of the most frequent splice junction were excluded. These junctions most likely originated from low-level transcripts. (ii) The 5' and 3' splice sites of all splice junctions were sorted in ascending order by their genomic position to determine exons and alternative splice sites. This was possible because 3' and subsequent 5' splice sites mark exon boundaries, whereas two consecutive 5' or 3' splice sites represent alternative splice sites. (iii) All explainable isoforms were determined based on determined splice junctions, exons and alternative splice sites. This was achieved by traversing from the first to the last exon via the splice junctions and initializing a novel independent isoform at each alternative 5' splice site and at splice junctions with the same 5' but a different 3' splice site. (iv) The set of putative isoforms was extended for isoforms showing retention of introns by analyzing the identified isoforms for occurrence of intronic regions. An intron was defined as retained if (a) all nucleotides of the intron were covered by at least one read and if (b) the average coverage of the intron was at least 10% of the average coverage of the adjacent exons. The retained introns were incorporated in the previous isoforms in all combinations, which led to a final set of intron free isoforms and isoforms containing different combinations of retained introns.

For quantification, the putative isoforms of each RNA-seq run were transferred into a Generic feature format version 3 (GFF3) file and, together with the corresponding HITSAT2 alignment file, used as input for Cufflinks (version 2.2.1, Trapnell et al., 2012). Cufflinks quantification was performed with default parameters except for the following modifications: –multi-read-correct and –GTF with the GFF3 file as input. The resulting FPKM values of all isoforms were afterwards transferred to relative abundance by dividing them by the FPKM value of the underlying gene, which corresponds to the sum of FPKM values of all gene isoforms.

### Promoter Analysis

For promoter analysis, the 1,000 bp DNA region upstream of the transcriptional unit was used for *cis*-element identification via PlantPAN v2.0 (Chow et al., 2016) using *A. thaliana* database. Heat shock elements (HSEs) were identified using the manual motif search with two or three consecutive palindromic nGANn or nGNAn on either + or – strand (Scharf et al., 2012), using the following input: GANNNNTC, GANNNTNC, GNANNNTC, GNANNTNC, NTCNNGAN, NTCNNGNA, TNCNNGAN, TNCNNGNA. Co-orthologs of A. thaliana transcription factors were identified as described for SRs. The transcript levels of the tomato transcription factors in response to HS, derived from an existing RNA-Seq dataset from *S. lycopersicum* cv. Moneymaker seedlings exposed to 25, 39 or 45°C (Hu et al., 2020a).

## Results

### Identification and Classification of *Solanum lycopersicum* SR Proteins

**Figure 1:**
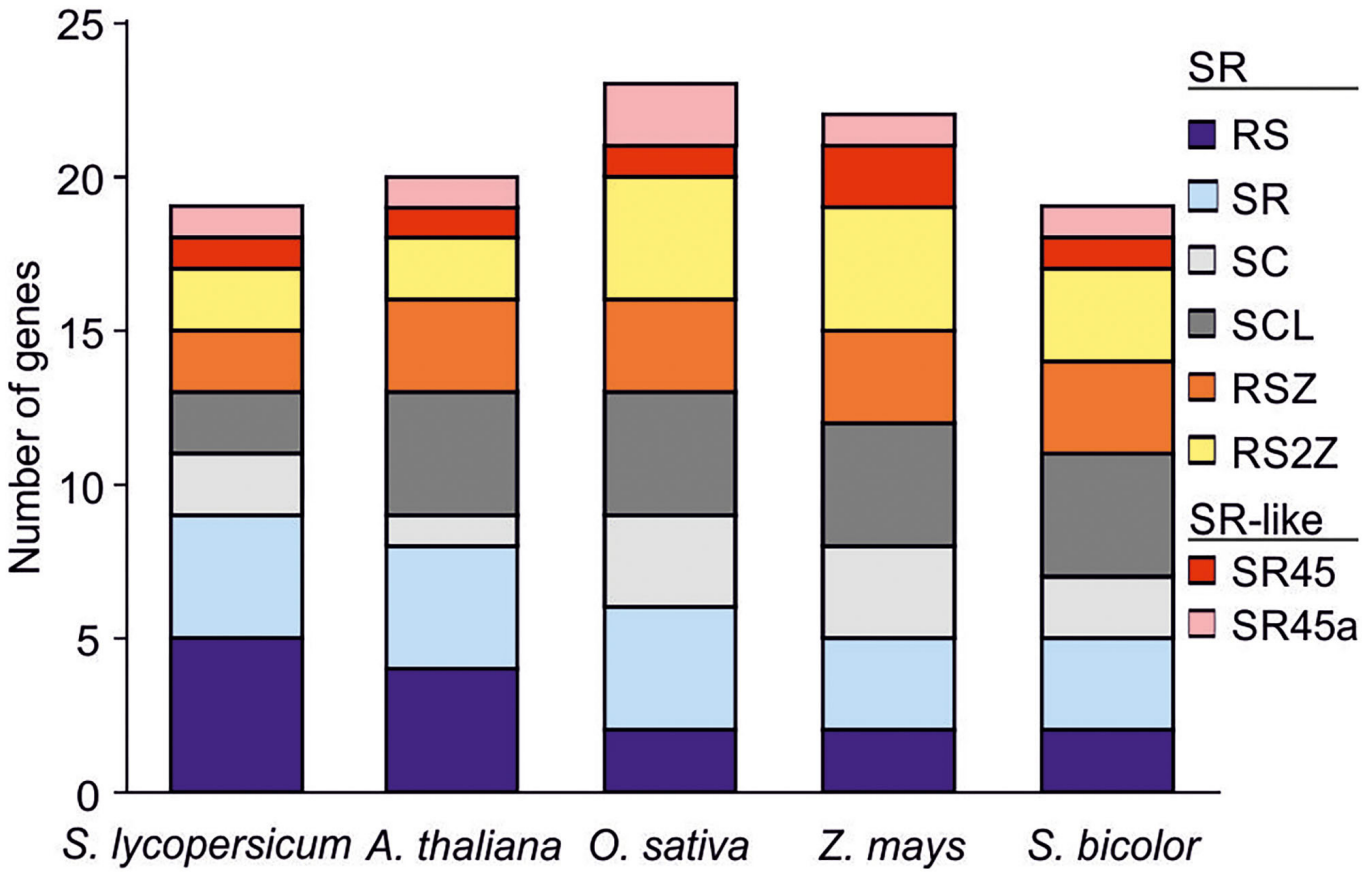
SR co-orthologs in different plant species. Co-orthologs of SR and SR-like proteins in tomato using previously characterized members from four plant species as bait (Tanabe et al., 2009; Richardson et al., 2011). Abbreviations are explained in text.

**Figure 2:**
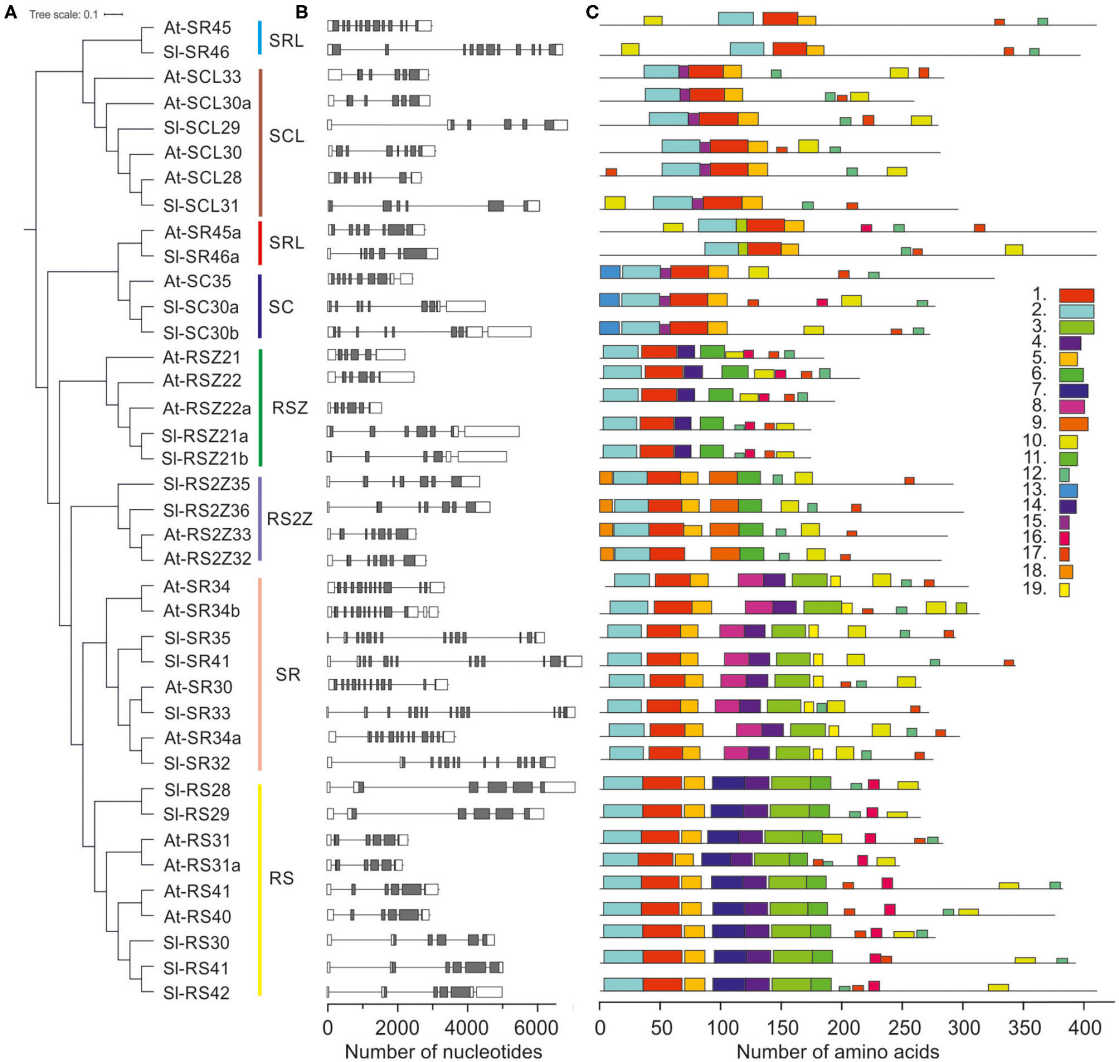
Gene structure and amino acid motif occurrence in the Arabidopsis and tomato SR protein gene family. **(A)** Unrooted neighbor joining tree. **(B)** Intron (line)/exon (boxes) distribution of SR coding genes. Gray and white boxes indicate coding and untranslated regions, respectively. **(C)** Motifs of SR proteins. Details on the sequence of each motif are provided in Supplementary Figure 1.

Fourteen SR co-orthologs of *A. thaliana, Oryza sativa, Sorghum bicolor* and *Zea mays* SRs (Chen, 2006; Östlund et al., 2009; Richardson et al., 2011) were identified in tomato (Figure 1; Supplementary Table 2). The gene inventory was supplemented with two genes annotated as “Arginine/serine-rich splicing factor” (Solyc10g009330, Solyc06g009060) in the tomato genome database (solgenomics.net). The identified SR proteins have one or two RRMs, but only 11 of them have an RS domain with an RS/SR dipeptide content higher than 20% as defined for plant SRs (Table 1; Barta et al., 2010). The proteins annotated as Sl-RS29, Sl-RS41, and Sl-RS42 have an RS/SR dipeptide content lower than 20%. However, all three belong to RS orthologous groups and share RS family motifs that also exist in *A. thaliana* co-orthologs (Figures 2A,C). Therefore, these three members are considered as canonical RS proteins. Two proteins are classified as SCL members based on the presence of the N-terminal charged extension, while four proteins with the SWQDLKD motif in the ΨRRM are assigned to the SR-subfamily (Figure 2C, Table 1).

**Table 1:** List of tomato SR proteins and position of their domains.

**Subfamily**	**Gene ID**	**Name**	**Protein (aa)**	**RRM position (aa)**	**ZnK position (aa)**	**RS/SR (%)**
RS	Solyc10g009330	Sl-RS28	239	4–61; 96–158		24
	Solyc01g096180	Sl-RS29	239	4–61; 95–157		8
	Solyc01g091750	Sl-RS30	250	4–63; 96–158		21.1
	Solyc11g072340	Sl-RS41	354	4–62; 97–159		12.4
	Solyc03g026240	Sl-RS42	370	4–62; 96–158		8
SR	Solyc03g082380	Sl-SR32	288	9–76; 117–179		63.4
	Solyc01g099810	Sl-SR33	285	8–75; 110–173		35.1
	Solyc09g075090	Sl-SR35	308	8–75; 114–176		48.5
	Solyc06g009060	Sl-SR41	360	8–75; 118–180		44.9
SC	Solyc04g074040	Sl-SC30a	258	18–87		29.4
	Solyc01g105140	Sl-SC30b	254	18–87		24.6
SCL	Solyc01g005820	Sl-SCL29	252	38–107		33.8
	Solyc01g080660	Sl-SCL31	267	41–111		26.4
RSZ	Solyc08g006435	Sl-RSZ21a	183	4–61	93–108	32.3
	Solyc08g069120	Sl-RSZ21b	183	4–61	93–108	30.8
RS2Z	Solyc05g054920	Sl-RS2Z35	306	13–73	103–118; 124–140	28.8
	Solyc09g005980	Sl-RS2Z36	315	14–75	104–119;125–142	56.9
SR-like	Solyc10g005590	Sl-SR46	416	116–184		52.8; 21.6
	Solyc06g076670	Sl-SR46a	389	50–120		32.7; 27.1

The inventory of tomato SRs was further used for a reciprocal best BLAST hit search in the tomato genome database (ITAG2.40) to identify putative SRs that were missed by the ortholog search approaches. The C-terminal region of Solyc08g006430, which is annotated as “N-methyl-L-tryptophan oxidase” shows high similarity to gene Solyc08g069120. The proposed full-length Solyc08g006430 open reading frame could not be amplified by RT-PCR, but transcripts were obtained when oligonucleotides for the 3'-region of the gene were used (data not shown). Therefore, we conclude that the Solyc08g006430 accession includes two genes, an N-methyl-L-tryptophan oxidase and the SR protein assigned to the Solyc08g006435 accession. Solyc08g006435 was annotated as Sl-RSZ21a and Solyc08g069120 as Sl-RSZ21b (Table 1).

The 17 SRs are distributed into six designated subfamilies, namely four SR, five RS, two SC, two SCL, two RSZ, and two RS2Z proteins (Figures 1, 2A; Table 1). Compared to *A. thaliana, Z. mays, O. sativa* and *S. bicolor*, the tomato genome encodes a lower number of SCL but higher number of RS co-orthologs (Figure 1). In addition, the co-orthologs of the well-characterized SR-like proteins At-SR45 (Ali et al., 2007) and At-SR45a (Tanabe et al., 2009) were identified, namely Sl-SR46 (Solyc10g005590) and Sl-SR46a (Solyc06g076670), respectively. Members of the same SR protein subfamily, from both tomato and Arabidopsis, have a similar number of introns. Notably, the genes of the SR subfamily contain more introns than genes of the other subfamilies (Figure 2B). However, compared to Arabidopsis, tomato SR genes generally possess longer introns (Figure 2B).

Sequence analysis of Arabidopsis and tomato SR proteins yielded between 6 and 11 motifs for each protein and 19 in total (Figure 2C). The motifs are either conserved among all members (e.g., RRM1, RRM2), or serve as signature sequences for specific subfamilies, e.g., motif 7 and 8 for RS and SR subfamilies, respectively (Figure 2C; Supplementary Figure 1). Motif 13 corresponds to the N-terminal extension of the SC subfamily. The ZnK motif 6 is present in RSZ and RS2Z subfamilies, whereas the ZnK motif 9 is present in RS2Z only (Figure 2C; Supplementary Figure 1).

### Expression Analysis of SR Genes

**Figure 3:**
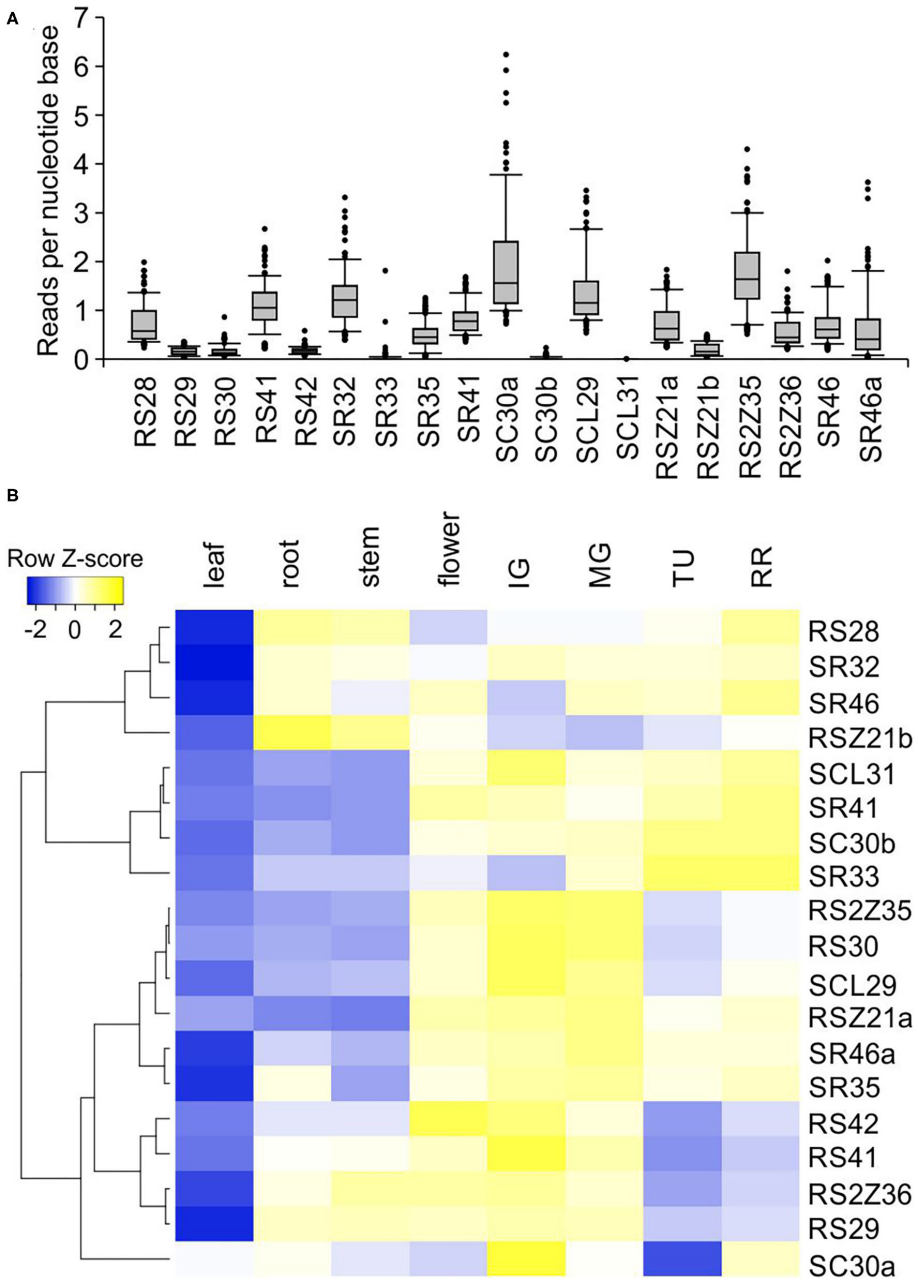
Expression of SR protein genes in different tomato organs and tissues. **(A)** Global expression profiles of each SR across 106 samples. Quantile boxplots (0.25, 0.75) show the distribution of the log2 transformed reads per nucleotide base normalized values obtained from TOMEXPRESS database (*n* = 106; Zouine et al., 2017). **(B)** Heat map depicting the relative transcript abundance of tomato SR genes in selected tomato organs based on qRT-PCR analysis. The dendrogram was generated by hierarchical clustering of genes based on Pearson distance of z-score data and average linkage using heatmapper (Babicki et al., 2016). –ΔΔCt values after normalization with CAC and EF1α housekeeping genes and leaf as calibrator sample were used. In **(A)** RSZ21a transcript levels correspond to Solyc08g006430; in **(B)** gene specific oligonucleotides for Solyc08g006435 amplification were used. Fruit stages: IG, immature green with 50% diameter of the mature green; MG, mature green; TU, turning; RR, red ripe.

Global transcript abundance profile analysis of SR coding genes from an existing set of 106 RNA-seq libraries including various tissues and organs revealed a variation in expression among SR genes. *Sl-RS30, Sl-RS42, Sl-SR33, Sl-RS29, Sl-RSZ21b, Sl-SC30b*, and *Sl-SCL31* are only weakly expressed in tomato tissues and organs, while others such as *Sl-SC30a* and *Sl-RS2Z35* show variable transcript levels (Figure 3A; Supplementary Dataset 1).

The total transcript levels of SR and SR-like genes were examined by qRT-PCR in different tomato organs and across fruit development. A preferential expression in specific organs and fruit ripening stages was observed (Supplementary Dataset 1). *Sl-RS28, Sl-SR32*, and *Sl-SR46* are enhanced in roots, stems, flowers and fruits compared to leaves (Figure 3B). *Sl-SCL31, Sl-SR41, Sl-SC30b*, and *Sl-SR33* are more abundant in flowers and fruits compared to vegetative organs, and enhanced during fruit ripening (Figure 3B). *Sl-RS2Z35, Sl-RS30, Sl-SCL29, Sl-RS2Z21a, Sl-SR46a*, and *Sl-SR35* are also more abundant in flowers and fruits compared to vegetative tissues but are gradually decreased during ripening. *Sl-RS42, Sl-RS41, Sl-RS2Z36, Sl-RS29*, and *Sl-SC30a* show enhanced levels in all tissues when compared to leaves, but peak in immature green fruits and are gradually reduced during ripening. These results suggest possible tissue/organ-preferential activity for some SR proteins.

### Alternative Splicing of SR Protein-Coding Genes

The existence of splice variants for SR and SR-like coding genes was determined by analysis of existing RNA-seq libraries from root, red ripe fruit and pollen tissues (Tomato Genome Consortium, 2012; Keller et al., 2017). This analysis generated high numbers of putative transcripts, reaching up to 43 for *Sl-SR33* (Supplementary Dataset 2). However, 39 out of the 43 *Sl-SR33* variants are only weakly expressed. Therefore, only variants with >5% abundance in at least one RNA-seq library were considered for further analyses. Nevertheless, a full list of all identified splice variants is provided in Supplementary Dataset 2.

**Figure 4:**
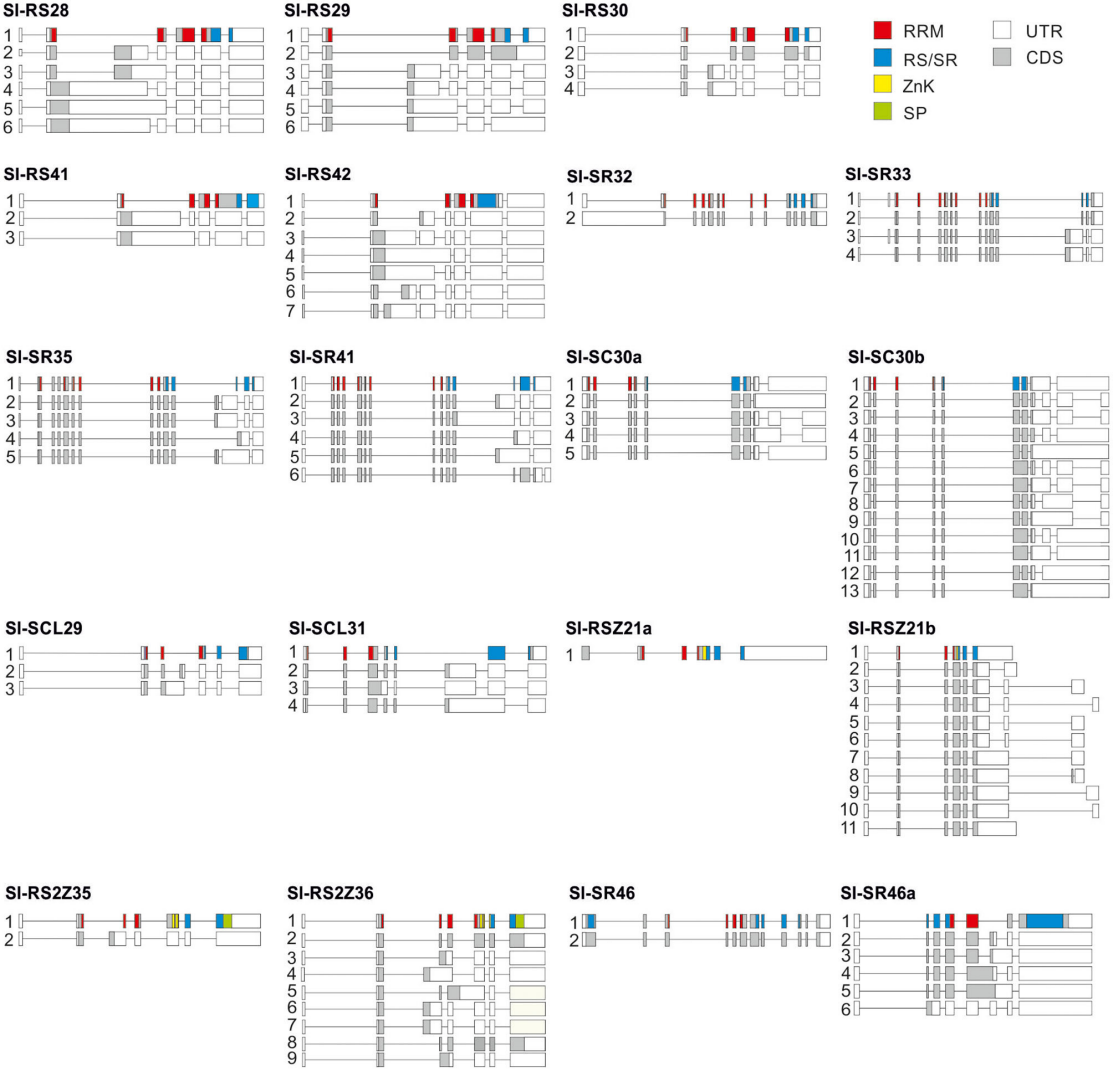
Gene models and splice variants of tomato SR and SR-like coding genes. Isoforms were identified by analysis of RNA-Seq databases from roots, red ripe fruits and pollen (Supplementary Dataset 2). Boxes indicate exons. Translated regions are colored and untranslated white. Color coding for domains is indicated on the upper right.

*Sl-RS2Z35* and *Sl-SR32* show the lowest complexity among the alternatively spliced genes, with each having only two transcript variants according to our selection criteria (Figure 4). In contrast, *Sl-SC30b* and *Sl-RS2Z36* show higher complexity with 13 and 9 variants, respectively (Figure 4). While *Sl-RSZ21b* shows a variable 3'-UTR due to alternative splicing, alternative spliced variants were not observed for *Sl-RSZ21a* in the indicated tissues (Figure 4).

Most of the SR genes possess a single transcript encoding for the full-length protein (Figure 4). However, alternative splicing in the 5'- or 3'-UTR results in multiple mRNAs coding for the full-length SR protein, as in the case of *Sl-SC30a* with five and *Sl-SC30b* with seven variants (Figure 4). In addition, some alternative splicing events cause minor in-frame sequence changes, as for example the inclusion of a Ser residue in the RS domain of Sl-SR46.2, Sl-RS2Z36.2, and Sl-RS2Z36.8 isoforms, the inclusion of a Gln in Sl-RS30.2 or the deletion of an Arg residue in the RS domain of Sl-SC30a.5 (Figure 4). Alternative splicing also results in an ~50% RS domain truncation in several isoforms of Sl-SR35 and Sl-SR41 (Figure 4).

All members of the SR, RS, and RS2Z subfamily as well as *Sl-SCL29* and *Sl-SR46a* contain an intron in their 5'UTR region (Figure 4). However, alternative splicing in the 5'-UTR intron was detected only for *Sl-SR32* and *Sl-SR33* (Figure 4). Furthermore, SC and RSZ members as well as *Sl-RS42* contain at least one intron in their 3'-UTR. Alternative splicing occurs in this region in both *Sl-SC30a* and *Sl-SC30b* (Figure 4).

Based on the RNA-seq analysis of the three tissues, for the majority of SRs, pollen AS profiles differ significantly from roots and fruits (Supplementary Figure 2). For example, *Sl-SC30a.1* and *Sl-RS42.1* are the major transcripts for the respective genes in roots and fruits, but are the least abundant in pollen (Supplementary Figure 2). To examine the variation in the levels of the protein-coding transcripts of SR genes across different tomato tissues, RT-PCR was performed on roots, stems and leaves from 6 week old plants, as well as flowers and fruits from various stages of development from 2 to 3 month old plants. The oligonucleotides were chosen to discriminate between spliced variants of the open reading frame (Supplementary Figure 3). Transcripts coding for isoforms with minor amino acid differences are considered as protein-coding. In the case of Sl-SR33, Sl-SR35, and Sl-SR41, transcripts coding for full-length proteins and isoforms with truncated RS domain were discriminated.

**Figure 5:**
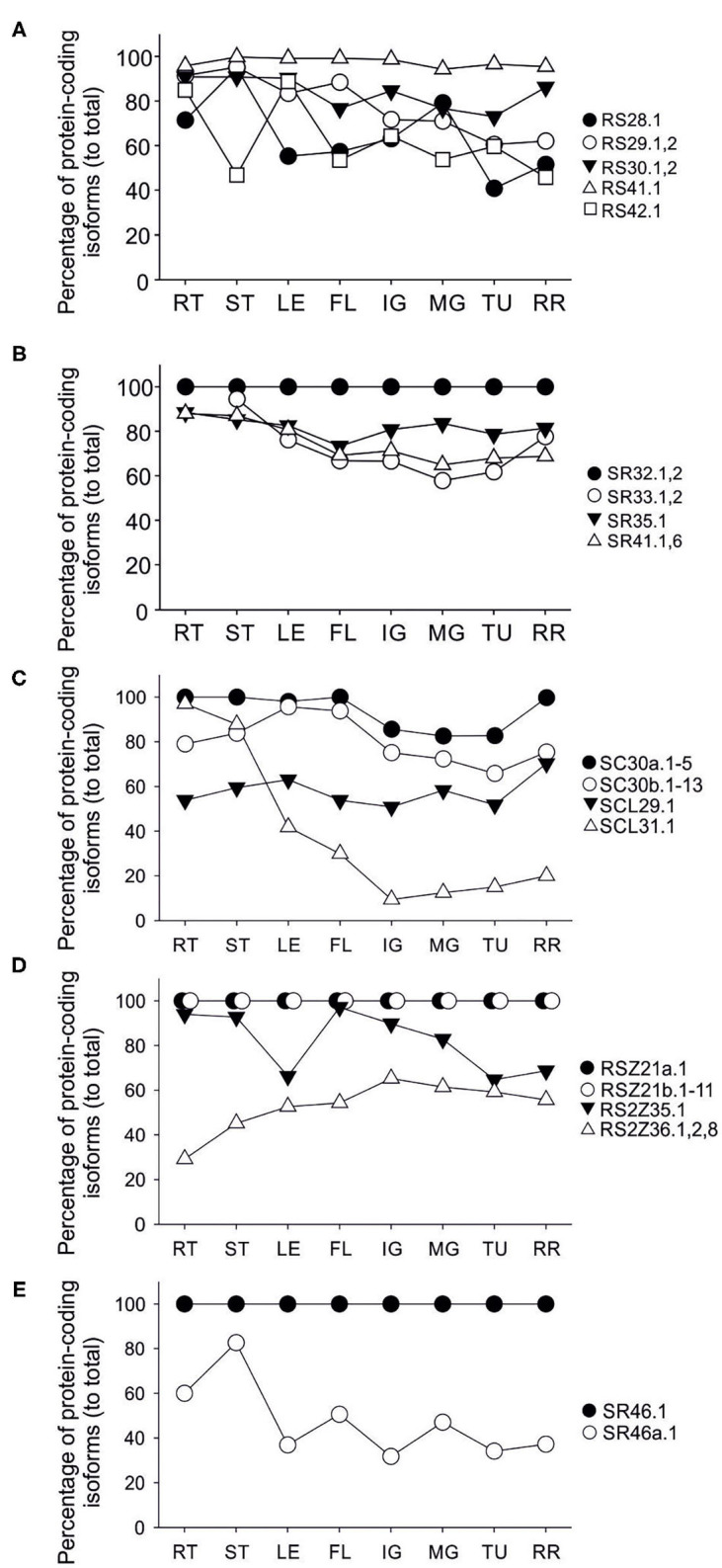
Transcript levels of protein-coding isoforms of SR and SR-like genes in tomato tissues. The average of relative transcript levels of protein-coding isoforms of indicated genes in root (RT), stem (ST), leaf (LE), flower (FL), and in fruit stages immature green (IG), mature green (MG), turning (TU) and red ripe (RR) are shown. Transcripts were analyzed by RT-PCR using oligonucleotides flanking the sites of alternative splicing within the open reading frame (Supplementary Figure 3) and quantified by densitometry using ImageJ 1.53 (Schindelin et al., 2012) after agarose gel electrophoresis and ethidium bromide staining. **(A)** RS, **(B)** SR, **(C)** SC and SCL, **(D)** RSZ and RS2Z, **(E)** SR-like genes.

No splice variants were detected for *Sl-SR32, Sl-RSZ21a, RSZ21b*, and *Sl-SR46* in any of the analyzed tissues (Figures 5A,D,E). Others such as *Sl-RS41, Sl-SC30a*, and *Sl-SC30b* are spliced but show no or very slight variation in the relative levels of their protein-coding transcripts throughout the samples (Figures 5A,C).

The protein-coding *Sl-RS28.1* transcript is enhanced in stem and MG fruit while those of *Sl-RS29 and* the full-length protein-coding *Sl-SR33* transcripts are reduced during fruit ripening (Figures 5A,B). For Sl-SR33 a reduction indicates an accumulation of the variants coding for the truncated protein isoforms. *Sl-SCL31* protein-coding transcripts show a dramatic reduction in all fruit stages when compared to other tissues (Figure 5A). *Sl-SR41* full-length protein-coding transcripts are also reduced during fruit ripening suggesting a relative increase in the levels of the transcripts coding for the putatively truncated isoforms (Figure 5B). While the levels of the protein-coding *Sl-RS2Z35* levels are lower in leaf and advanced stages of fruit ripening those of *Sl-RS2Z36* are higher in all fruit stages when compared to root, suggesting that alternative splicing has a different impact on the two members of the subfamily (Figure 5D). *Sl-SR46a.1* transcript accumulated at higher levels in root and stem compared to the other tissues (Figure 5E).

### Changes in the Splicing Profile and Transcript Levels of SR Coding Genes Under Heat Stress

The splicing profile of many genes is altered under HS conditions (Keller et al., 2017; Ling et al., 2018). Thus, the impact of HS treatment on the splicing efficiency of SR genes was explored, which might result in enhanced or reduced fraction of protein-coding transcripts when compared to transcripts derived from non-productive alternative splicing. The latter are likely targeted to NMD. For this, RT-PCR was performed using oligonucleotides that anneal in the regions flanking the alternative splicing sites within the open reading frame, as previously described (Supplementary Figure 3). Protein-coding transcripts are considered as well as those predicted to code for isoforms with minor changes, while in the case of Sl-SR33, Sl-SR35, and Sl-SR41, full-length and truncated protein-coding transcripts are discriminated. However, an effect on splicing profile of these three SR genes was not observed. A representative agarose gel for each SR gene can be found in Supplementary Figure 4. The fraction of protein-coding transcripts is further referred to as splicing efficiency.

**Figure 6:**
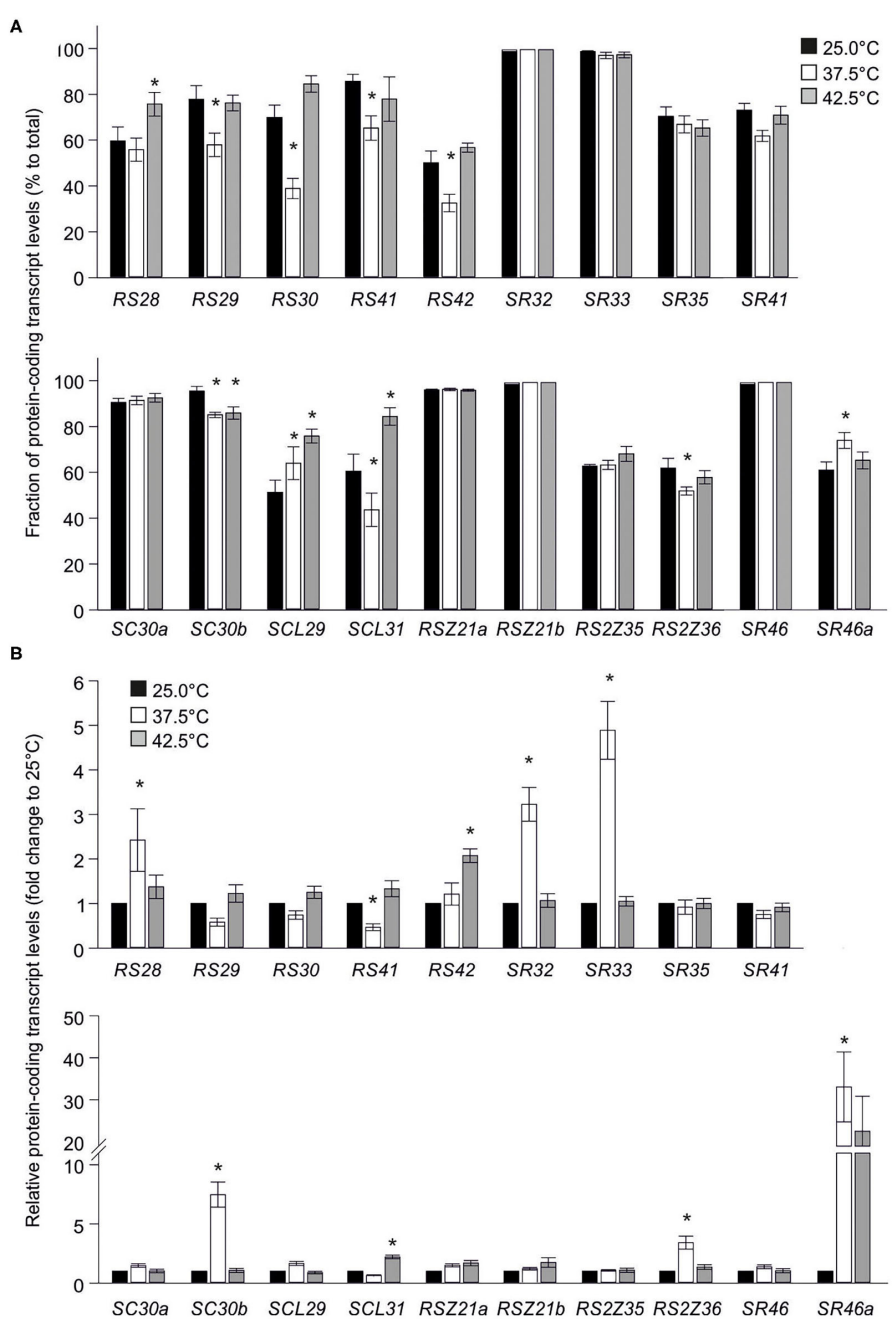
Effect of elevated temperatures on transcript levels of tomato SR and SR-like coding genes. **(A)** Levels of SR and SR-like protein-coding transcripts in tomato leaves exposed to 25, 37.5 or 42.5°C for 1 h, relative to total transcripts. Transcript levels were determined as in Figure 5. **(B)** Relative levels of transcripts of SR protein-coding isoforms in young leaves of tomato plants exposed to 25, 37.5 or 42.5°C for 1 h. The levels of heat stressed samples are expressed as relative to the control (25°C) sample (1-fold) and calculated with the 2^−ΔΔCt^ method (Livak and Schmittgen, 2001) using EF1α as control. Values are the average of six independent biological replicates and asterisks indicate statistically significant difference to control (>2-fold change and *p* < 0.05 based on ANOVA with Duncan *post hoc* test).

Significant changes in the splicing efficiency were detected for eight SR and one SR-like genes in heat stressed samples when compared to control. The splicing efficiency of *Sl-RS29, Sl-RS30, Sl-RS41, Sl-RS42, Sl-SCL31*, and *Sl-RS2Z36* is reduced in leaves exposed to 37.5°C when compared to control, while that of *Sl-SC30b* is reduced in leaves exposed to either 37.5 or 42.5°C (Figure 6A). In contrast, the relative levels *Sl-SCL29* protein-coding transcripts are induced under both HS temperatures, while those of *Sl-SR46a* are induced only in 37.5°C treated samples and those of *Sl-RS28* are induced only in 42.5°C treated samples (Figure 6A). Interestingly, while the splicing efficiency of *Sl-SCL31* is reduced in leaves exposed to 37.5°C, it is enhanced in leaves exposed 42.5°C.

Further, the levels of the protein-coding transcripts of the SR and SR-like genes under HS conditions were compared to control. qRT-PCR analysis on leaves treated the same way as for splicing analysis revealed that *Sl-RS28, Sl-SR32, Sl-SR33, Sl-SC30b, Sl-RS2Z36*, and *Sl-SR46a* protein-coding transcripts are enhanced in response to a 37.5°C treatment when compared to 25°C (Figure 6B). *Sl-SCL31* and *Sl-RS42* protein-coding transcripts are upregulated only in leaves exposed to 42.5°C. Only *Sl-RS41* shows a reduction in transcript levels in 37.5°C treated leaves (Figure 6B). The enhanced levels of protein-coding transcripts of several SRs do not coincide with their splicing profile and therefore is rather pointing to a transcriptional regulation.

**Figure 7:**
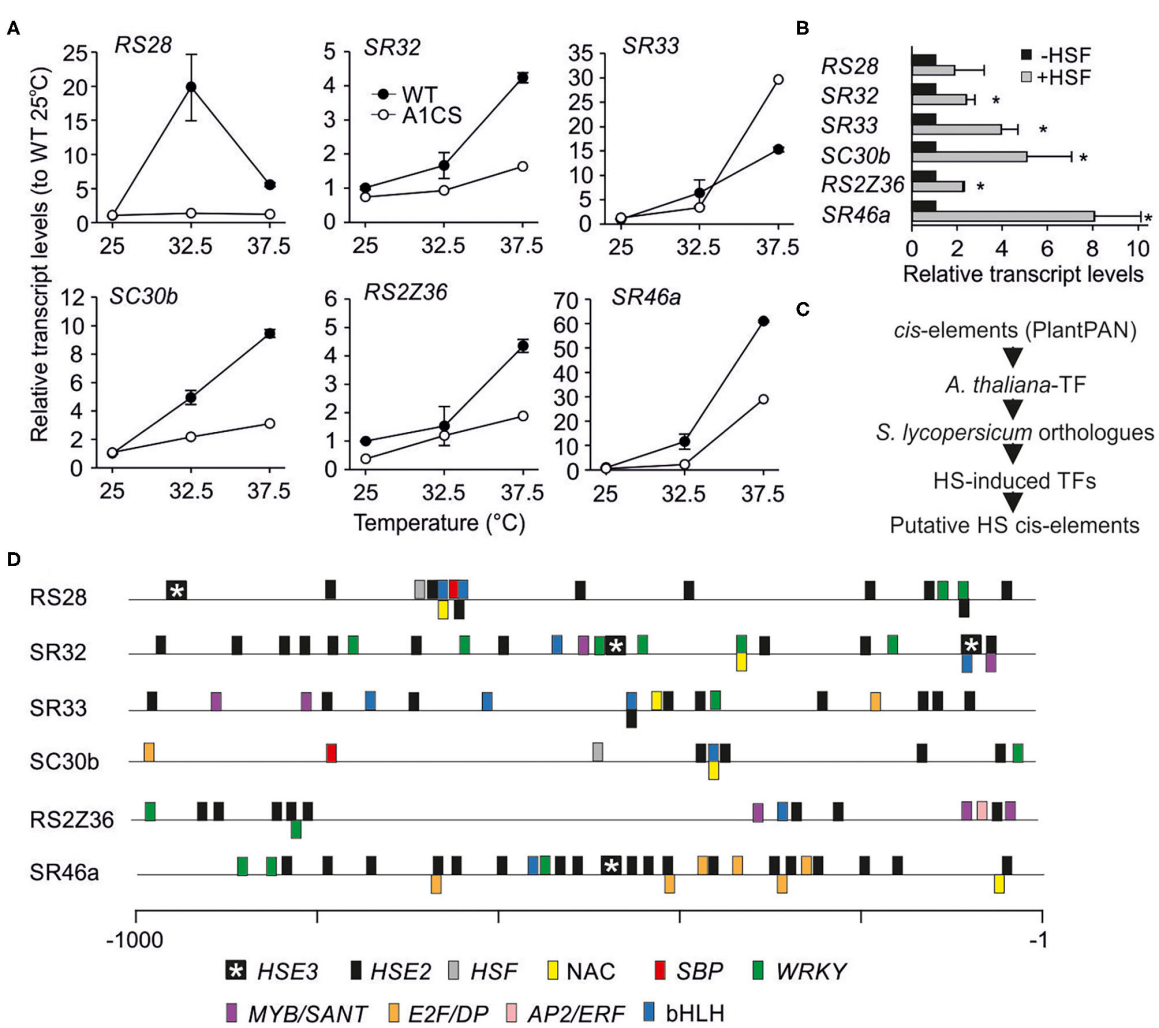
Regulation of selected SR genes under heat stress. **(A)** Total transcript levels of the indicated SR coding genes in wild type and A1CS young leaves exposed to 25, 32.5 or 37.5°C for 1 h. Relative levels were calculated as in Figure 6B. **(B)** Relative levels of total transcripts of the indicated endogenous SR genes in protoplasts co-expressing HsfA1a and HsfA2 (+HSF). Relative levels were calculated as in **(A)** using mock protoplasts (–HSF) as control sample. Each value is the average of 3 independent replicates and error bars indicate ±SE. Asterisks indicate significant difference (*p* < 0.05) compared to control based on pairwise *t*-test. **(C)** Workflow for the identification of *cis*-elements in SRs genes. **(D)** The relative position of the *cis*-elements identified in the promoter region of 1,000 bp upstream of the initiation codon for each of the indicated genes (Supplementary Dataset 4). The elements are denoted in color code. HSE2 and HSE3 are composed of two or three palindromic nGANn or nGNAn motifs.

The upregulation of the total transcripts of the six HS-induced genes was confirmed by qRT-PCR using RNA isolated from leaves of WT plants exposed to 32.5 or 37.5°C (Figure 7A). In tomato, HsfA1a is essential for the upregulation of the majority of HS-induced genes (Mishra et al., 2002) and thus, the co-suppression line was included in the analysis. All HS-induced SRs showed a reduced upregulation at 37.5°C in A1CS leaves when compared to WT, while the induction of *Sl-RS28* is suppressed entirely (Figure 7A). In contrast, *Sl-SR33* shows an even stronger upregulation in 37.5°C-treated A1CS plants compared to WT (Figure 7A).

HsfA1a and HsfA2 build activator complexes to stimulate the induction of HS-related genes (Scharf et al., 1998). Therefore, we checked whether the expression of HsfA1a and HsfA2 in tomato mesophyll protoplasts under control conditions leads to the induction of selected SR genes. With the exception of *Sl-RS28*, the transcripts of all other examined genes were induced in HsfA1a/HsfA2 expression protoplasts further supporting the active involvement of HSFs on SR regulation (Figure 7B).

The presence of putative *cis-*elements in the promoters of all HS-induced SR genes was analyzed. As currently a comprehensive *cis*-element database for tomato is not available, the *A. thaliana* PlantPAN v. 2.0 database was used (Figure 7C). 477 matrix IDs and motifs were identified as binding sites for 615 *A. thaliana* transcription factors (Supplementary Dataset 3). These correspond to 399 co-orthologs in tomato. Only 22 of these 399 TFs are HS-induced based on an available transcriptome analysis of different tomato cultivars exposed to HS (Supplementary Dataset 4; Hu et al., 2020a).

At first, the presence of HS elements (HSEs) in the promoters of all HS-induced SR genes was analyzed. All HS-induced SR genes contain HSEs with two consecutive palindromic nGANn or nGNAn motifs (HSE2) as minimal requirement for HSF binding. In addition, Sl-RS28, Sl-SR32, and Sl-SR46a also contain HSEs with three motifs (HSE3; Figure 7D) that are thought to be bound by trimeric HSFs (Scharf et al., 2012). The regulation by HSFs is in line with the presence of HSEs in the promoters of SR genes. Moreover, Sl-SR46a with the highest number of HSEs was the most strongly induced gene under HS.

Further, the existence of additional promoter elements was determined, because an HSF-independent accumulation of the transcript in response to HS was observed. Indeed, elements recognized by at least one of the 22 HS-induced TFs could be identified in the promotor regions. Among them are binding sites for MYB, NAC, bHLH and WRKY transcription factors that might stimulate the expression of SRs under HS (Figure 7D; Supplementary Dataset 4).

### Effect of High Temperatures on SR Protein Abundance

**Figure 8:**
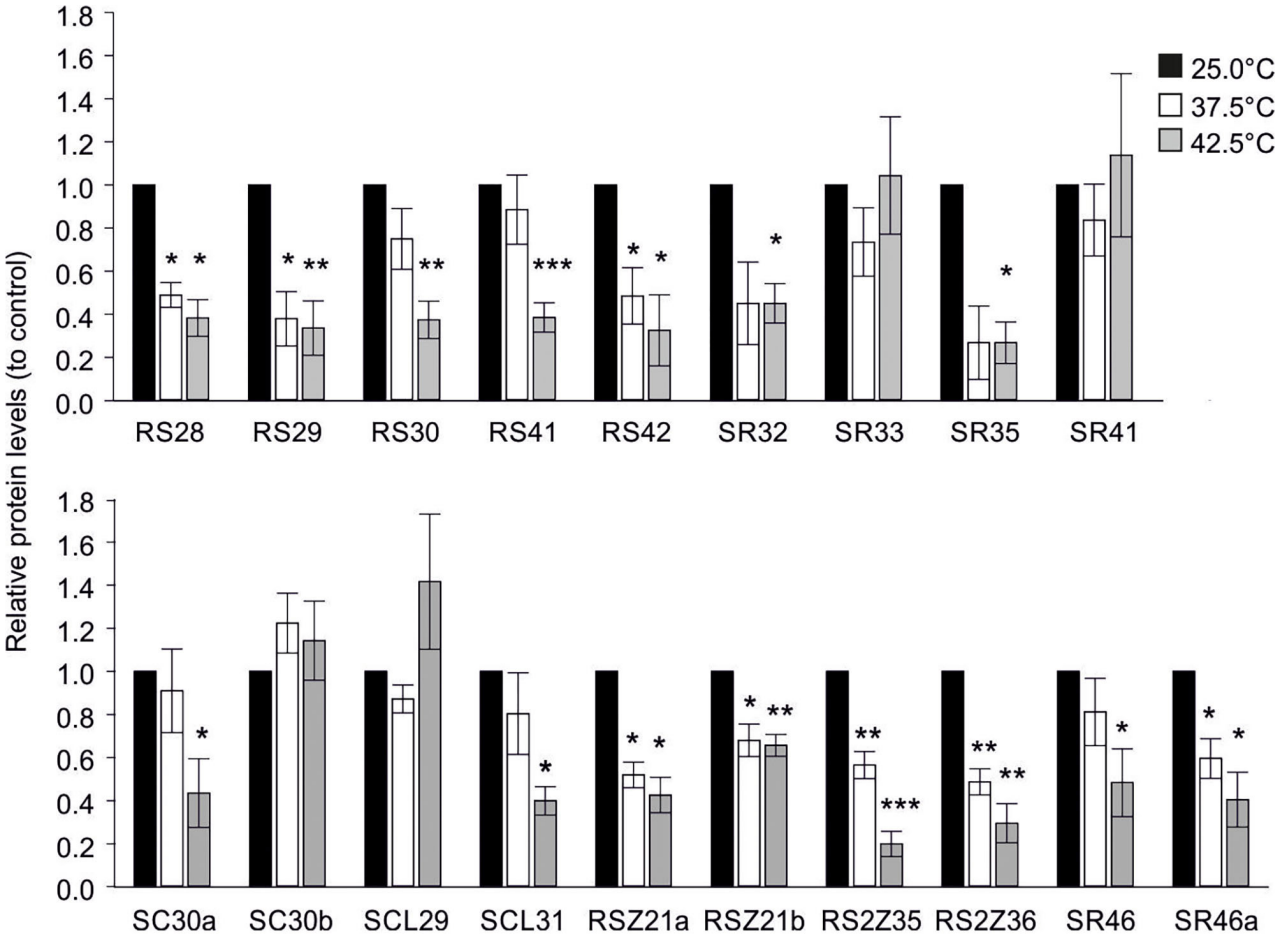
Effect of high temperatures on protein abundance of HA-tagged SR proteins in tomato protoplasts. HA-tagged SR and SR-like proteins were expressed in tomato protoplasts for 5 h, followed by exposure to the indicated temperatures for 1 h. Protein levels were analyzed by quantification of signals using ImageJ 1.53 (Schindelin et al., 2012), following immunodetection with αHA antibody. Values are the average of 3 independent experiments with error bars indicating ±SE. For each experiment, the signal intensities were first normalized against the endogenous Hsc70 and then to the 25°C sample. Asterisks indicate statistically significant difference between treated (37.5 or 42.5°C) and control sample (25°C) based on paired *t*-test comparison (*p < 0.05, **p < 0.01, ***p < 0.001). Representative immunoblots can be found in Supplementary Figure 5.

High temperatures have a significant impact on proteome integrity (Goldberg, 2003). Consequently, the effect of increased temperatures on the abundance of HA-tagged SR proteins expressed in tomato mesophyll protoplasts was examined by immunoblot analysis. Following transfection with the respective expression plasmids carrying the CDS coding the full-length protein for each gene under the control of the CaMV 35S promoter, protoplasts were incubated at 25°C for 5 h to allow protein accumulation and then subsequently exposed to 37.5 or 42.5°C for 1 h, or kept at 25°C as control (Figure 8). The experiment was repeated 3 independent times, and representative immunoblots can be found in Supplementary Figure 5.

The majority of SRs showed a reduction in protein levels under both stress treatments when compared to the control, including all members of the RSZ and RS2Z subfamilies as well as Sl-RS28, Sl-RS29, Sl-RS42, Sl-SR46a (Figure 8). Interestingly, HA-tagged Sl-RS30, Sl-RS41, Sl-SR32, Sl-SR35, Sl-SC30a, Sl-SCL31, and Sl-SR46 levels were only significantly affected by the 42.5°C treatment (Figure 8). In contrast, Sl-SR33, Sl-SR41, Sl-SC30b, and Sl-SCL29 show steady state levels under both stress treatments (Figure 8). Therefore, these results highlight significant differences in temperature-dependent protein abundance even among members of the same subfamily.

## Discussion

### Regulation of Tomato SR Protein-Coding Genes in Different Tissues

Alternative splicing is an important mechanism that expands proteome diversity, but also contributes to the regulation of proteome abundance under both physiological and stress conditions (Filichkin et al., 2015). Tissue-specific splicing has been shown for tomato ovule and pericarp tissues (Wang et al., 2016), while splicing complexity is higher during early fruit growth when compared to seedlings and flowers (Sun and Xiao, 2015). This process is tightly regulated, and the occurrence of variations in alternative splicing suggests the existence of specific factors for different spliceosome compositions. The factors involved in these processes need to be identified in order to understand the control of cellular and organismic processes related to growth, development and stress response.

We identified 17 SR protein-coding genes in tomato, and two additional SR-like proteins as co-orthologs of SRs from other plant species (Figure 1). The total number of SR genes is similar to that of *A. thaliana* and *S. bicolor* (Reddy et al., 2012; Chen et al., 2019). Interestingly, the tomato genome encodes for a higher number of RS but a lower number of SCL genes, compared to the other species examined here (Figure 1) or to the recently published SR inventory of the moss *Physcomitrella patens* (Melo et al., 2020). Such differences might be related to species-specific requirements for RNA regulation, as for example during tomato fruit development (Sun and Xiao, 2015). In support of this notion, several RS-coding genes are highly expressed in early stages of fruit growth and development (Figure 3).

The differential expression of SR genes indicates that except the expected functional redundancy that has been already demonstrated in some cases in *A. thaliana* (Cruz et al., 2014; Xing et al., 2015; Carvalho et al., 2016; Yan et al., 2017), a developmental stage and tissue preferential functions might exist as well (Figure 3). Many SR coding genes, such as *Sl-RS2Z35, Sl-RS30*, and *Sl-SCL29*, are expressed at higher levels in immature green fruits undergoing cell expansion, which is in agreement with a higher demand for factors to support pre-mRNA processing during fruit development. While these genes show reduced levels in advanced stages of fruit ripening, others are upregulated, including *Sl-SCL31, Sl-SR41, Sl-SC30b*, and *Sl-SR33* (Figure 3). These results support the hypothesis on the existence of a dynamic regulatory network that shape developmental stage specific splicing events.

We also observed variations in the transcript levels among SR genes belonging to the same subfamily, with at least one member being expressed at generally higher levels than the other(s) in various tissues (Figure 3). Interestingly, the generally low expressed *Sl-SC30b* and *Sl-RS2Z36* are upregulated by HS, which is not the case for the more abundant *Sl-SC30a* and *Sl-RS2Z35* (Figures 3, 6). This indicates that the low expressed SRs might be functionally relevant under specific environmental conditions or even in specific cell types.

The majority of SR genes undergo alternative splicing themselves (Isshiki et al., 2006; Palusa et al., 2007; Yoon et al., 2018; Chen et al., 2019; Melo et al., 2020). Indeed, with the exception of *Sl-RSZ21a*, all other SR and SR-like genes are alternatively spliced (Figure 4). The absence of alternative splicing in at least one RSZ gene has been reported in *A. thaliana, B. rapa*, and *Manihot esculenta* (cassava) (Palusa et al., 2007; Yoon et al., 2018; Gu et al., 2020). Moreover, the conservation in alternative splicing between Arabidopsis and rice SR genes, for example in the introns flanking the RRM coding region (Kalyna et al., 2006), is confirmed here for tomato (Figure 4). In most of the cases, alternative splicing in tomato RS genes leads to putatively NMD-targeted transcripts as for Arabidopsis and rice. In contrast, alternative splicing in members of the SR subfamily in both Arabidopsis (Palusa et al., 2007) and tomato occurs in the introns spanning the exon encoding the RS domain coding region, resulting most likely in protein isoforms with truncated RS domains (Figure 4). Thus, SR splicing is largely conserved across different plant species.

The majority of alternative splicing events in tomato SR genes lead to the generation of aberrant transcripts (Figure 4) which are likely targeted for NMD as shown in Arabidopsis (Palusa and Reddy, 2015). Other alternative splicing events contribute to variations in the 5'- or 3'-UTR. However, alternative splicing events that lead to C-terminal truncations or putative protein isoforms with minor amino acid sequence differences in the RS domain exist as well (Figure 4; Supplementary Dataset 2). Such changes can generate isoforms with different functions. Consistent with this notion, the two isoforms of At-SR45, which differ by an insertion of eight amino acids in At-SR45.1 that replaces an arginine in At-SR45.2, complement different developmental phenotypes in the *at-sr45* mutant (Zhang and Mount, 2009). Moreover, only At-SR45.1 is involved in plant tolerance against salinity stress (Albaqami et al., 2019).

The splicing efficiency of the introns within the coding region of nine SR genes showed a variation in splicing efficiency among different tomato tissues larger than 20%. Remarkably, *Sl-SCL31* shows a high splicing variation from 97% in roots to 9% in immature green fruits (Figure 5). In addition, five genes show mild changes of <20% in the relative levels of the protein-coding transcripts. In general, the splicing variation is slightly higher in fruits and flowers compared to vegetative organs, which is in agreement with splicing variation observed by analysis of RNA-seq samples from root, red fruit and pollen (Supplementary Figure 2). A higher variation in the splicing profile for many SRs in pollen compared to other tissues has been also shown in Arabidopsis (Palusa et al., 2007).

Changes in splicing efficiency correlate with transcript levels for some SRs, e.g., a gradual reduction in splicing efficiency in *Sl-RS29* coincides with a similar reduction profile in transcript levels (Figures 3, 5). The positive relation between splicing efficiency and transcript profile among different tissues is observed for *Sl-SR33* as well. However, an opposite profile was detected as well. For example, while the splicing efficiency of *Sl-RS28* is reduced during fruit ripening, the transcript levels are enhanced. Such differences suggest that both transcriptional regulation and alternative splicing contribute to the abundance of SRs in tomato tissues.

### SR Genes Are Regulated at Multiple Levels in Response to High Temperatures

Exposure to high temperatures has a prominent effect on pre-mRNA splicing of many genes (Filichkin et al., 2010, 2015; Keller et al., 2017; Ling et al., 2018). Consistent, HS treatments affects the splicing efficiency of several SR coding genes causing an ~10–30% alteration in the relative levels of the full-length protein-coding transcripts (Figure 6). Among all SRs, only *Sl-RS28, Sl-SCL29, Sl-SCL31*, and *Sl-SR46a* show enhanced splicing under one or both HS treatments. Instead, six SR genes show reduced splicing efficiency at 37.5°C but not at 42.5°C. The latter is surprising as a more severe stress is expected to have a stronger inhibitory effect on pre-mRNA splicing, as for example shown previously for the HS-induced HsfA2 (Hu et al., 2020b). We can envision that the activity of splicing silencers that reduce intron splicing at 37.5°C for several SRs, might be suppressed or deactivated under stronger temperatures. Vice versa, the temperature-dependent activation of splicing enhancers and/or the suppression of splicing silencers probably contributes to the increased splicing efficiency of *Sl-RS28, Sl-SCL29, Sl-SCL31*, and *Sl-SR46a* (Figure 6). We assume that the regulation of SR-coding genes further allows the manipulation of HS-sensitive alternative splicing.

While reduced levels of transcripts encoding for full-length Sl-RS41 under HS can be attributed to reduced splicing efficiency, *Sl-SC30b* and *Sl-RS2Z36* show reduced splicing efficiency but enhanced transcript levels. The total transcript levels of both these genes, along with *Sl-RS28, Sl-SR32, Sl-SR33*, and *Sl-SR46a* are induced in response to mild HS, suggesting that transcriptional regulation is the major driver for the accumulation of their full-length protein-coding transcripts.

The induction of S*l-SR32, Sl-SC30b, Sl-RS2Z36*, and *Sl-SR46a* is reduced in A1CS leaves exposed to HS, suggesting that HsfA1a stimulated their accumulation but is not required for their induction (Figure 7). The induction of SRs in protoplasts expressing HsfA1a-HsfA2 co-activator complexes and the presence of HSEs in their promoter regions argues for an HSF-dependent transcriptional regulation including *Sl-SR33* (Figure 7).

*Sl-SR33* shows a reduced accumulation at 32.5°C in A1CS leaves compared to WT but an even stronger upregulation in A1CS leaves treated with 37.5°C. This might point to an HSF-mediated positive and negative regulation at different temperatures. While the positive regulator HsfA1a can stimulate the induction of *Sl-SR33* under mild conditions, as supported by the upregulation of this gene in HsfA1a-HsfA2 expressing protoplasts, under higher temperatures the activity of an HsfA1a-dependent repressor like HsfB1 might contribute to its suppression (Mishra et al., 2002; Fragkostefanakis et al., 2018). Interestingly, some HSFs, including HsfA2, are also subjected to temperature sensitive alternative splicing (Hu et al., 2020b) and therefore we can assume the existence of a feedback mechanism between SR and HSF genes.

In the promoters of the SR-genes, *cis*-elements that form putative binding sites for HS-induced tomato transcription factors exist (Figure 7). These factors include, among others, members of the WRKY, MYB, NAC, and bHLH gene families. We assume that a cooperation between these factors and HSFs could cause the transcriptional stimulation of selected SR genes under high temperatures. The presence of these *cis*-elements might indicate that these SRs are also induced by other abiotic stresses. The ortholog of NAC protein Solyc11g017470 in *A. thaliana* (AT1G01720) is induced after a treatment with abscisic acid (ABA) and is involved in abiotic stress responses (Jensen et al., 2013). An ABA-dependent regulation has been shown for several Arabidopsis and cassava SRs (Cruz et al., 2014; Gu et al., 2020) and therefore, considering that ABA is involved in HS response, we can assume an interplay of different regulatory networks to control SR transcription under high temperatures. However, this hypothesis needs to be addressed experimentally in the future.

At the protein level, tomato SR members show different sensitivity to high temperatures, as some sustain steady-state levels in heat stressed protoplasts, while others are reduced even under mild HS conditions (Figure 8). We assume that changes in protein abundance most likely reflect the effect of temperatures on protein stability, as in several cases a reduction in protein levels did not coincide with reduced transcript levels of the endogenous transcripts under HS, as for example in Sl-RS2Z35 and Sl-RS2Z36 (Figure 8; Supplementary Figure 4). Moreover, the observed differences in protein abundance are not likely due to an effect on transcription, as all SRs are expressed under the control of the CaMV 35S promoter. This is also supported by the fact that some SRs show steady state levels while some are reduced. Therefore, although different levels of regulation might contribute to changes in protein abundance of SRs, we propose that protein stability plays a dominant role. Based on these results, we assume that differences in protein abundance contribute to changes in the alternative splicing profiles of target genes in heat stressed cells. In some cases, the increased sensitivity of SR proteins under HS might be compensated by an induction of transcripts, as in the case of *Sl-RS2Z36, Sl-SR32, Sl-RS28*, and *Sl-SR46a* (Figures 6, 8). Transcriptional induction of heat resistant SRs such as *Sl-SR33* and *Sl-SC30b* could lead to an even higher protein accumulation (Figures 6, 8).

In conclusion, our results show that both transcriptional regulation and alternative splicing contribute to the abundance of SRs in tomato tissues. Splicing of SR genes is largely conserved across different plant species. This process generates isoforms with different sequences and thus, likely different functions. The different SR proteins and isoforms are likely part of a dynamic regulatory network that shape developmental stage specific and stress response splicing events. On the one hand, several tomato SR protein-coding genes are differentially expressed in different organs. On the other hand, transcription of SRs is dependent on environmental conditions and at least in part HSF dependent. This places SRs into the network of HS regulation, which is supported by the observed alternative splicing of HSFs. Thus, the regulatory network between SR and HSF might be a feedback loop. Moreover, splicing of SRs is temperature sensitive as well, balanced likely by temperature sensitive splicing enhancers and silencers. Future attempts to decipher the role of the individual SRs in temperature responses will offer insights into their specific contribution in shaping the transcriptome landscape in tomato.

## Data Availability Statement

The datasets presented in this study can be found in online repositories. The names of the repository/repositories and accession number(s) can be found in the article/Supplementary Material.

## Author Contributions

RR did the majority of the experiments with the assistance of SB and SV. SS did the orthology analysis. MK did the RNA-seq data analysis. SF conceptualized and headed the project with the help of ES. SF, ES, and RR wrote the manuscript, which was read and approved by all authors.

## Conflict of Interest

The authors declare that the research was conducted in the absence of any commercial or financial relationships that could be construed as a potential conflict of interest.
