# Supplementary material for: Identification and Regulation of Tomato Serine/Arginine-Rich Proteins Under High Temperatures
Source: Front Plant Sci. 2021 Mar 29;12:645689. doi: 10.3389/fpls.2021.645689 (PMC8039515; doi:10.3389/fpls.2021.645689)
Supplement: Supplementary file 3 [file Data_Sheet_5.DOCX]

Supplementary Material

# Supplementary Data

**Supplementary Dataset 1**. Transcript levels of SR and SR-like tomato genes across tomato tissues and organs based on TOMEXPRESS RNA-seq database.

**Supplementary Dataset 2**. Splice variants of tomato SR and SR-like coding genes and their transcript levels in tomato tissues based on RNA-seq analysis.

**Supplementary Dataset 3**. List of heat stress elements and *cis*-elements putatively bound by heat stressed induced transcription factors.

**Supplementary Dataset 4**. List of Arabidopsis transcription factors and their tomato heat stress induced orthologues with putative binding sites in promoters of heat stress induced SR and SR-like coding genes.

# Supplementary Figures and Tables

## Supplementary Figures

**Supplementary** Figure 1. MEME motifs in amino acid sequence of Arabidopsis and tomato SR and SR-like proteins.

**Supplementary Figure 2**. Relative transcript levels of SR gene transcript variants in root, red ripe fruit and pollen from RNA-seq datasets.

**Supplementary Figure 3**. Annealing positions of oligonucleotides used for PCR.

**Supplementary Figure 4**. Splicing profile of SR coding genes in response to heat stress. The splice variants for each gene are indicated on the right. Asterisk indicates protein-coding transcript. EF1a was used as reference gene.

**Supplementary Figure 5**. Representative immunoblots of HS-tagged SR proteins expressed in protoplasts which were exposed to 25, 37.5 or 42.5°C for 1 hour.

## Supplementary Tables

**Supplementary Table 1**. Oligonucleotides used in this study.

**Supplementary Table 2**. Orthologues of plant SR and SR-like protein-coding genes.

## Supplementary Figures

**
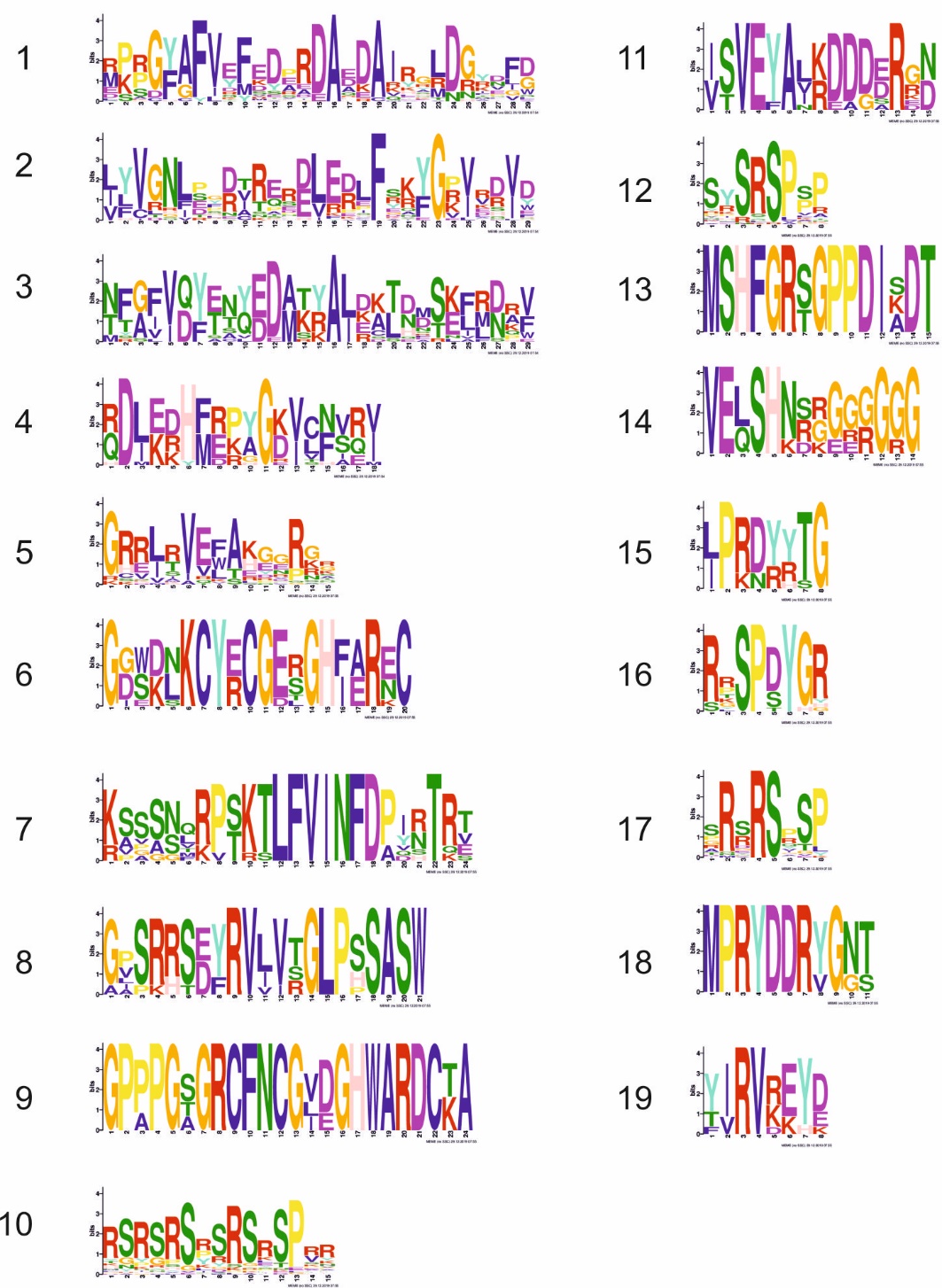
**

**Supplementary** Figure 1. MEME motifs in amino acid sequence of Arabidopsis and tomato SR and SR-like proteins.

**
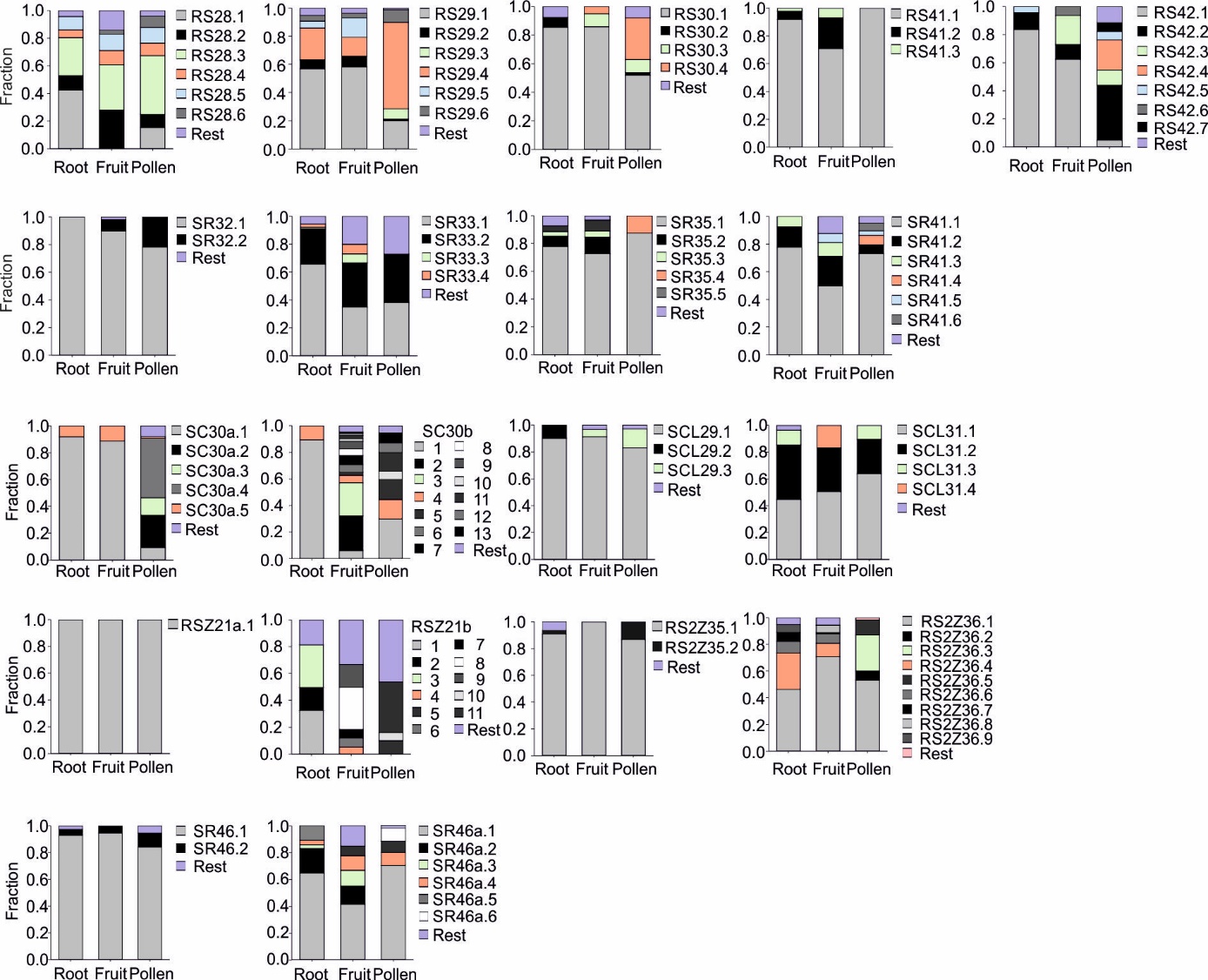
**

**Supplementary Figure 2**. Relative transcript levels of SR gene transcript variants in root, red ripe fruit and pollen from RNA-seq datasets.

**
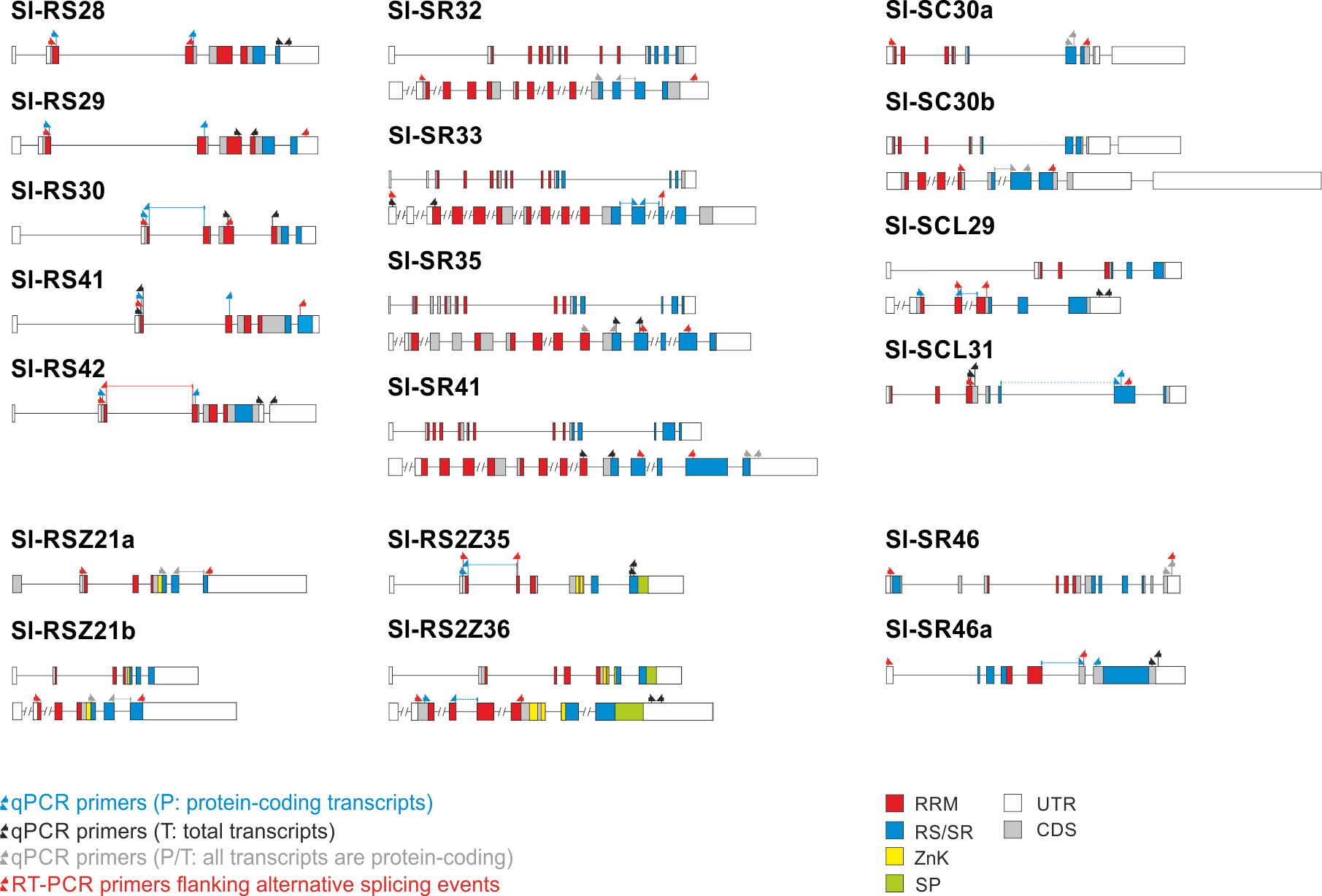
**

**Supplementary Figure 3**. Annealing positions of oligonucleotides used for PCR.

**
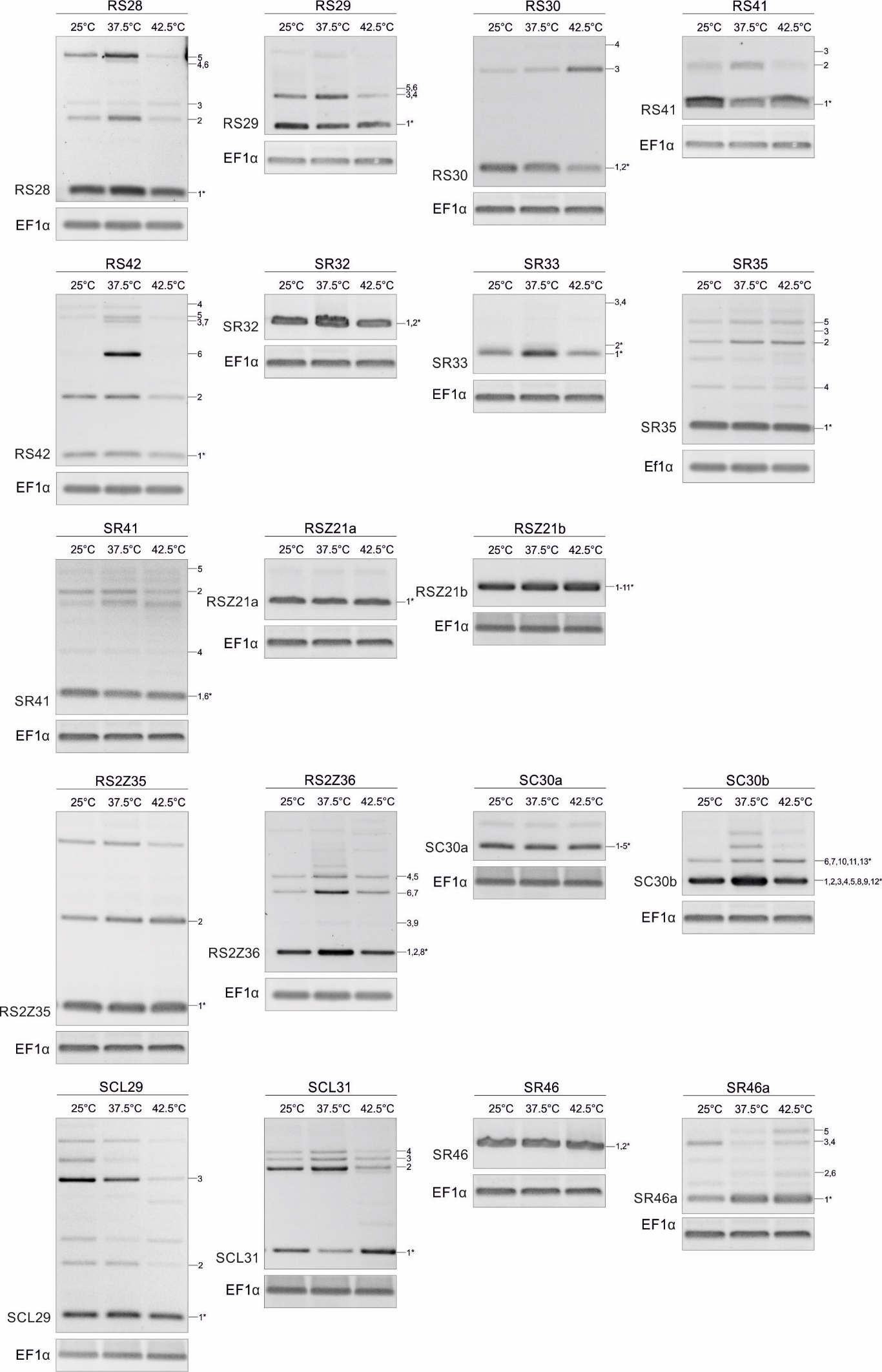
**

**Supplementary Figure 4**. Splicing profile of SR coding genes in response to heat stress. The splice variants for each gene are indicated on the right. Asterisk indicates protein-coding transcript. EF1a was used as reference gene.

**
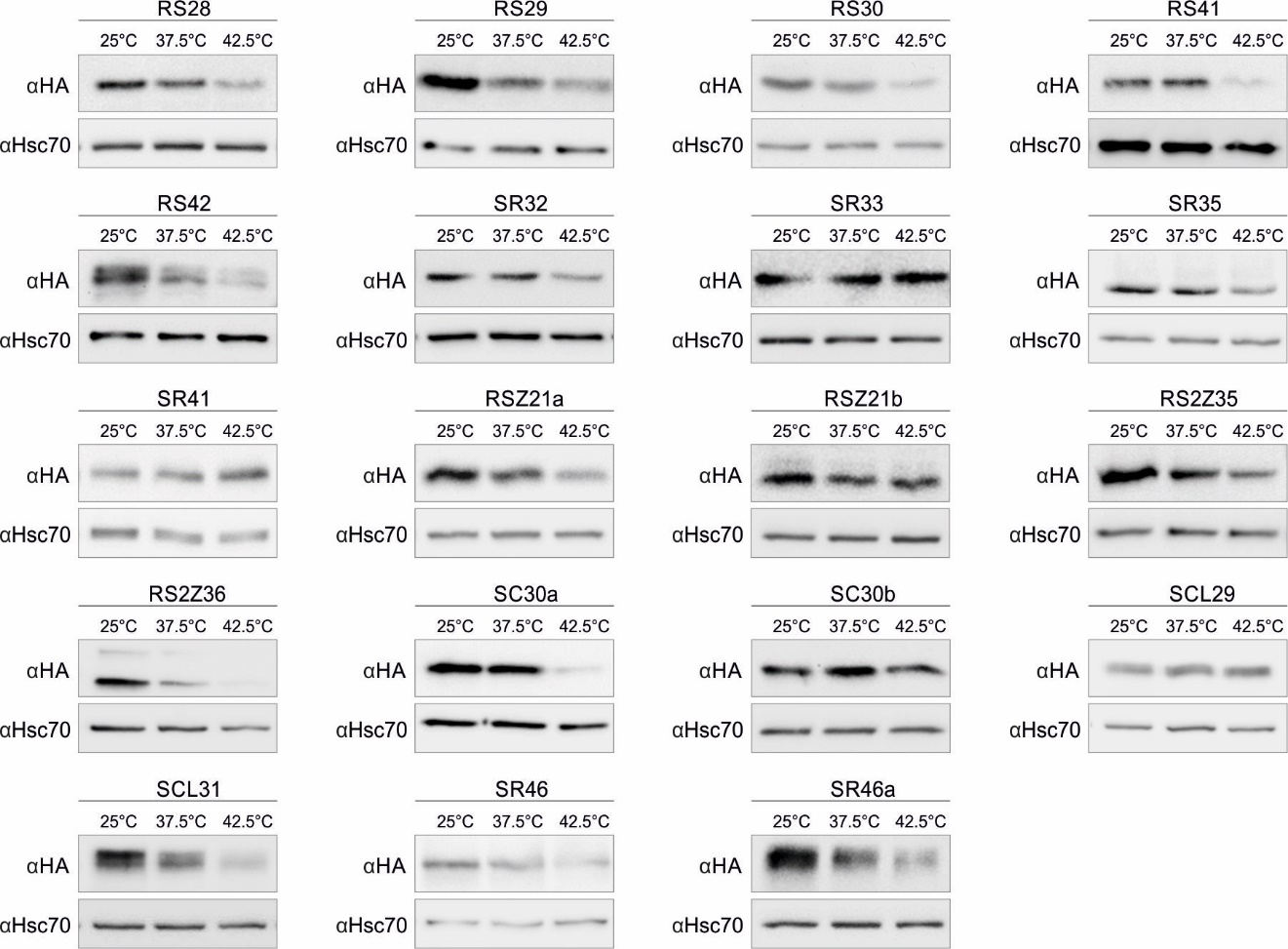
**

**Supplementary Figure 5**. Representative immunoblots of HS-tagged SR proteins expressed in protoplasts which were exposed to 25, 37.5 or 42.5°C for 1 hour.
